# Human safety of SPBN GASGAS for oral rabies vaccination of dogs

**DOI:** 10.1371/journal.pntd.0013866

**Published:** 2026-01-12

**Authors:** Gowri Yale, Conrad Freuling, Andrew Gibson, Amila Gunesekera, Thomas Müller, Ryan Wallace, Ad Vos

**Affiliations:** 1 Global Rabies Program, Ceva Santé Animale, Libourne, France; 2 Institute for Molecular Virology and Biology, Friedrich-Loeffler-Institut, Greifswald, Germany; 3 Mission Rabies, Worldwide Veterinary Services, Dorset, United Kingdom; 4 Rabies Treatment Unit, National Hospital, Colombo, Sri Lanka; 5 Poxvirus and Rabies Branch, Centers for Disease Control and Prevention, Atlanta, Georgia, United States of America; The University of Kansas, UNITED STATES OF AMERICA

## Abstract

Oral rabies vaccination (ORV) of dogs has been suggested as a complementary tool to injectable rabies vaccination, to access free-roaming dogs during mass dog vaccination campaigns. Stringent safety requirements have been developed and implemented for ORVs targeting wildlife and dogs. Part of the safety requirements involves conducting a risk assessment of the potential for human contact with the vaccine virus and the impact on human health. The former is predominantly determined by the bait distribution method and the latter by the safety profile of the vaccine construct. This risk assessment is focused on a commercially available ORV for dogs, SPBN GASGAS. Overall, it is concluded that although human contacts with the vaccine virus do occur infrequently, the risk of serious adverse events in humans is negligible due to the safety profile of the vaccine virus construct.

## Introduction

Oral rabies vaccination (ORV) of wildlife populations has resulted in a paradigm shift in wildlife disease management [1,2]. Initially targeted at red foxes, the concept has been adapted to many other reservoir species, including raccoons, gray fox, coyotes, jackals, and raccoon dogs. ORV has led to the elimination of rabies from many targeted areas in Europe, North America, and Asia [3]. In view of the historical successes of ORV in wildlife and the more recent Tripartite WHO/WOAH/FAO “zero by 30” initiative to eliminate dog-mediated human rabies deaths by the year 2030, the use of ORV in dogs is now recommended as a tool for rabies management and elimination [4,5]. The recommendation for the use of ORV as a complement to injectable vaccines targeting dog populations is reflected in the recent guidance document from the tripartite “Oral vaccination of dogs against rabies” [6]. Vaccination approaches integrating ORV are especially impactful in situations where a large proportion of dogs are free-roaming and not easily accessible for vaccination by the traditional injectable route [7,8].

This manuscript provides the first comprehensive synthesis linking laboratory, regulatory, and field safety data for SPBN GASGAS, highlighting its compliance with new WHO/WOAH/FAO (2023) recommendations.

While historical use of ORVs in wildlife had been conducted using a wide variety of vaccine virus constructs with different safety profiles [9], the co-habitation of people and dogs has resulted in heightened attention to the safety risk assessments and distribution methods of ORV to dogs [4] (S1 Table). Prior vaccine safety modeling studies showed that early first and second-generation ORVs that were used in wildlife could pose a risk, albeit very rare, to human safety if they were used in dogs [10]. Among the various ORVs developed and deployed over the past decades, SPBN GASGAS has emerged as a third-generation vaccine that overcomes critical limitations of earlier constructs. It is a genetically engineered derivative of the SAD (Street Alabama Dufferin) strain, featuring multiple safety-enhancing modifications that prevent reversion to virulence, resulting in a superior safety profile. The modern technologies used to attenuate these vaccines showed that this third-generation vaccine now has a near-negligible risk if used in free-roaming dog populations [11,12]. Furthermore, rabies transmission models suggested that the only way to eliminate dog-mediated rabies in most low- and middle-income countries will require the use of oral vaccines [7]. With the recent convergence of factors supporting ORV of dogs, including advancements in vaccine safety technology, robust evidence-based studies demonstrating the necessity of ORV to eliminate dog-mediated rabies, and endorsement from the Tripartite, here we present a standardized safety assessment of one commercially available ORV (SPBN GASGAS). This assessment aims to determine if this product complies with international safety guidance [13] and dispel any remaining reservations.

## Risk assessment

A structured risk assessment aims to evaluate the likelihood and severity of serious adverse events (SAE), identify potential points of risk, and assess the impact of possible mitigation strategies [14] This manuscript presents a comprehensive narrative review of the safety data available for SPBN GASGAS, a licensed third-generation ORV, in key target species including foxes, raccoon dogs, and domestic dogs.

Although this work followed a narrative review format, a structured approach was applied to ensure completeness and transparency. Literature was searched in PubMed and Google Scholar for the period 1995–2024, using combinations of the terms “oral rabies vaccine,” “SPBN GASGAS,” “safety,” “risk assessment,” and “genetic stability.” Studies were included if they contained information on vaccine safety, stability, environmental risk, or field performance in target or non-target species. As the objective is to provide an overview of existing evidence and support informed decision-making regarding vaccine use, a formal systematic review or meta-analysis was not deemed appropriate for the current scope. In the context of rabies management strategies using ORV, one of the primary concerns is public health and safety, making the risk of SAEs in humans the central focus of this assessment [6]. Although not a part of this assessment, animal health is also a concern, and this vaccine has been extensively tested in domestic animals and wildlife species and found to be safe [15,16].

A comprehensive risk assessment should address the key SAE risks for humans, following standard environmental risk assessment methodologies. It should evaluate major safety concerns for humans using established guidelines, such as those from the European Medicines Agency, to assess the perceived risks associated with the release of such products [17]. As every rabies management action carries some level of risk, these risks will be categorized as high, moderate, low, or negligible. This categorization enables a rational, risk-benefit-based decision on the use of ORV as a tool to control and ultimately eliminate dog-mediated human rabies.

## SPBN GASGAS vaccine

**Fig 1:**
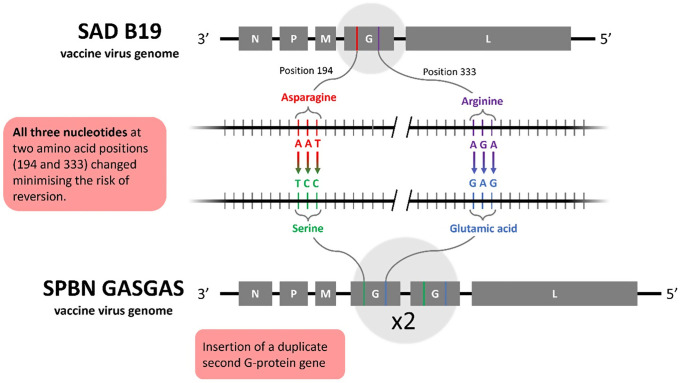
Schematic depiction of the targeted modifications in the genome of the SAD B19 vaccine virus (top) resulting in the SPBN GASGAS vaccine virus with a duplicate of the double-mutated glycoprotein (bottom). The genetic modifications in the RNA encoding for the amino acids at position 194 and 333 of the glycoprotein are enlarged (middle) showing the original codons (in red and violet) and the mutated codons (in green and light blue).

The SPBN GASGAS vaccine virus was developed from SAD L16, a cDNA clone of the attenuated rabies vaccine virus strain, SAD B19. Over 250 million doses of SAD B19 have been distributed since 1983, significantly contributing to the elimination of fox rabies in large parts of Europe. SPBN GASGAS was created by modifying the glycoprotein gene of SAD L16 at two key codon positions (333 and 194) and then inserting a duplicate of this modified gene into the genome (Fig 1). The first modification changed the codon at position 333, known to influence rabies virus pathogenicity, from encoding Arginine (AGA) to Glutamic Acid (GAG). This change resulted in the SPBN GA virus, which is further attenuated and non-pathogenic in mice. However, a compensatory mutation occurred at position 194 during serial passaging, which was preemptively addressed by changing Asparagine (AAT) to Serine (TCC) [18]. The final SPBN GASGAS construct, with a duplicate glycoprotein gene, enhanced safety (no reversion to virulence) by promoting apoptosis of infected cells and improving virus clearance [19]. In short, SPBN GASGAS was developed through multiple targeted mutations to the genome of an already attenuated rabies vaccine virus strain which further minimized pathogenicity, as well as risk of regaining pathogenic potential through subsequent mutation [20].

## Safety in target and non-target species

The ORV SPBN GASGAS has been rigorously evaluated to ensure minimal risk to target and non-target species. Non-target exposure primarily occurs when species other than the intended target species population, such as domestic or wild animals, consume vaccine baits. Extensive safety studies for SPBN GASGAS have been conducted in both target (dog, fox, raccoon dogs, mongoose) and non-target species (cat, field mouse, guinea pig, house mouse, raccoon, striped skunk, swine) [15,16,21] (Table 1) in addition to the safety studies already conducted for the less attenuated parent SAD B19 strain (brown rat, cattle, ferret, magpie, marten, mink, non-human primate-chimpanzee and baboon, pigeon, sibling vole, wood mouse [22] following international guidelines [23]. No SAEs were observed during these studies with SPBN GASGAS evaluating among others the effects of overdose, repeated doses, and different routes of administration.

**Table 1:** Safety studies of SPBN GASGAS on Target and Non-Target species.

Target and Non-Target species	Intrinsic safety	Overdose/repeated dose	Dissemination within the vaccinated host	SAEs	Reference
Dog (*Canis familiaris*)	X	X	None	None	[15,21]
Cat (*Felis catus*)	X	X	None	None	[15,21]
Pig (*sus scrofa domesticus*)	X		None	None	[15,21]
Field mouse (*Apodemus sylvaticus*)	X		None	None	[15]
Fox (*Vulpes vulpes*)	X	X	None	None	[15,21]
Guinea pig (*Cavia porcellus*)	X		None	None	[15]
House mouse (*Mus musculus*)	X		None	None	[15]
Mongoose (*Herpestes javanicus*)	X		None	None	[16,21]
Raccoon (*Procyon lotor*)	X		None	None	[21]
Raccoon dog (*Nyctereutes procyonoides*)	X	X	None	None	[15,21]
Striped skunk (*Mephitis mephitis*)	X		None	None	[21]

Furthermore, no vertical or horizontal transmission and no dissemination of the vaccine virus within the vaccinated animal beyond the sites of entry occurred [15,16]. After uptake in the oropharyngeal cavity, esp. the palatine tonsils, transient presence of the vaccine virus in the peripheral layers is observed before elimination by the innate immune response [24]. Additionally, the vaccine virus was not transferred to offspring when mothers (red fox) were vaccinated during pregnancy and no SAEs were observed in the pregnant animals and their offspring [15]. The risks associated with the interactions of the vaccine virus with target and non-target species are therefore considered “negligible.”

## Immunocompromised individuals

The World Organisation for Animal Health (WOAH) recommends that ORVs be evaluated for safety in immunocompromised animal models too, such as nude and SCID (Severe Combined Immuno-Deficiency) mice [25]. In accordance with these guidelines, SPBN GASGAS was administered to both SCID and nude mice via direct oral administration (DOA), subcutaneous (SC) injection, and intracerebral (IC) inoculation [10]. In these studies, SPBN GASGAS was shown to be safe in SCID and nude mice when administered via direct oral administration (DOA) and subcutaneous (SC) injection.

However, as expected, it only caused disease when directly injected into the brain of severely immunocompromised individuals with only 58% of the mice succumbing to rabies and testing positive for rabies antigen, in contrast to SAD B19 all mice succumbed to rabies after IC administration (Head and colleagues [10]). Thus, the risks associated with residual pathogenicity of the vaccine strain are considered “negligible” as only severely immune-compromised hosts are susceptible after severe exposure.

## Genetic stability

Genetic stability of live replicating vaccines is crucial to understand, as widely used human vaccines have been associated with infections due to genetic reversion to virulence. A notable example is the oral polio vaccine which now causes more human polio cases than the wildtype but is still considered a net-benefit to human health [26]. SPBN GASGAS, like all replication-competent rabies viruses, can be prone to mutations due to the lack of proofreading in RNA virus polymerases. This makes genetic stability, particularly at modified codons, crucial to prevent reversion to its original form, SAD B19. To assess the genetic stability of SPBN GASGAS, researchers conducted five serial passages both in vivo and in vitro. In the production cell line BHK21 Cl13, the virus underwent five passages starting from the master seed virus, followed by full genome sequencing. Results showed that SPBN GASGAS remained genetically stable as no changes in the consensus strain including the critical safety modifications at codons 194 and 333 were observed [27].

Further testing involved back-passage of the virus in Suckling Mouse Brain (SMB), a model highly susceptible to neurotropic viruses like RABV. After five SMB passages, the virus was inoculated intracerebrally in adult mice, with no reversion to virulence observed. Genome sequencing confirmed the stability of both glycoprotein genes and the introduced safety mutations, even after multiple passages, ensuring the vaccine’s safety and effectiveness. As part of the manufacturing process, the purity and identity of every batch is checked prior to release. These tests include the confirmation that the targeted genetic modifications are present, and that the vaccine did not revert (partially) to the less attenuated parental strain SAD B19. It is concluded that the risk of reversion to virulence is “negligible.”

## Environmental safety

Rabies virus is notably short-lived in the environment, demonstrating mere hours of viability when exposed to direct UV light or dry conditions, making this a good candidate for a vaccine construct in relation to environmental safety. The potential for environmental spread of SBPN GASGAS virus was evaluated by studying viral shedding in vaccinated animals and the possibility of transmission to unvaccinated animals [15,16,21]. These studies found no active shedding of the vaccine virus in saliva, urine, or feces.

However, the vaccine virus can be detected only in saliva shortly after oral administration [21], meaning animals that consume a bait could potentially transmit the virus to others through biting or social grooming during this brief period before the virus is cleared from the oral cavity. Indirect evidence of horizontal transmission was observed in several studies where naïve animals housed with vaccinated animals tested positive for rabies antibodies [15]. Essentially, this demonstrates the potential for seroconversion (vaccination) in dogs near recently vaccinated dogs through contact. The risk associated with release (distribution) of the vaccine virus in the environment is considered “low” to “negligible,” depending also on the bait distribution system (see Vaccine Distribution System below).

## Human safety

Touching an intact vaccine bait is safe and not considered an exposure [6]. Human risks associated with ORV can arise in two ways, direct or indirect contact with the vaccine. The first part of the assessment focuses on the likelihood of human contact with the vaccine virus, while the second part evaluates the risks to human health related to such contacts with the SPBN GASGAS virus strain. For ORV to impact human health, individuals must first come into contact with the vaccine virus not just the intact vaccine bait. This can happen during or shortly after a campaign, either through direct contact with leaking vaccine baits or discarded vaccine sachets, or indirectly through contact with very recently orally vaccinated dogs.

In case of human exposure to SPBN GASGAS, the exposed person should seek medical advice and report the exposure to public health authorities. The treatment, if warranted, depends on the type of exposure. Referring to the Tripartite recommendation on ORV of dogs, for this particular hazard and vaccine strain, rabies post-exposure prophylaxis (PEP) is recommended only for immune-compromised persons with a Category III exposure (contact of mucous membranes or open skin wounds with the vaccine virus) or Category IV exposure (severe bites) due to a dog recently (<48 h) ingesting a vaccine bait containing SPBN GASGAS [6]. This 48 h contains a safety margin as no viable vaccine virus has been detected more than 24 h post vaccination in the saliva of the vaccinated dogs [21]. In other situations, people licked, scratched, or bitten by a dog who has recently consumed an oral vaccine bait, immediate flushing and washing of the areas of contact with soap and water is sufficient. Therefore, it is concluded that the risk for humans is ‘negligible’. In rabies endemic settings, WHO PEP guidelines [28] should also be followed after any exposure to dog bite or saliva regardless of the dog’s oral vaccination status (Table 2).

**Table 2:** Overall risk levels based on SPBN GASGAS safety assessments.

Category	Outcome SPBN GASGAS	Risk Level			
		Negligible^1^	Low^2^	Moderate^3^	High^4^
Safety in target species	Non pathogenic	X			
Safety in non-target species	Non pathogenic	X			
Safety in immunocompromised animals	Non pathogenic	X			
Genetic stability	Stable	X			
Environmental safety	No shedding, except for input vaccine virus		X		
Human safety	Safe, and mitigating measures (PEP) obliviate risk	X			
**Overall Assessment**		**X**			

^1^The likelihood of the event occurring is so small that it can be ignored or is practically zero.

^2^The event is possible but unlikely; consequences would be minimum or/and well-contained.

^3^The event could occur occasionally; or, though unlikely, the consequences could be serious

^4^The event is likely or expected to occur, and/or would have significant adverse consequences.

## Vaccine bait distribution system

A key component of a successful ORV program involves the delivery of the vaccine bait to the target population as efficiently as possible to avoid wastage and limit vaccine-contact to non-target species, including humans. Various ORV baiting strategies for dogs have been suggested; 1) hand-out and retrieve model, 2) distribution to dog owners, and 3) wildlife model [6]. The most suitable and effective distribution system depends on the local situation. The hand-out and retrieve model targets the local population of owned or unowned dogs that are difficult to reach with injectable vaccines [29]. Vaccine baits are offered directly to these dogs and any baits that are not consumed are retrieved by the vaccinators to minimize the risk of non-target exposure and reduce costs by distributing those retrieved vaccine baits.

In some areas, it can be very inefficient to systemically locate free-ranging dogs, especially in areas where the human and dog population are widely scattered. Under these circumstances, offering baits to the dog owners (at a central location) who subsequently will give the baits to their dogs could be an effective and efficient alternative. Finally, the wildlife model is especially suitable for those free-roaming dogs that typically avoid human contact and cannot be approached within a distance that allows the bait to be offered directly. Here, vaccine baits are placed at selected sites or distributed across targeted habitats to more effectively reach dogs who will locate and consume the baits themselves without vaccinators being present. The risk that non-target species, incl. humans, can have direct contact with vaccine baits is of course lesser for the handout model and retrieve model than the wildlife model; “low” risk for the wildlife model and “negligible” risk for the handout model.

## Discussion

Oral vaccination is the preferred method for controlling and locally eliminating wildlife-mediated rabies in Europe and North America, leading to the declaration of large areas as rabies-free, except for bat rabies. Since 1978, over 1 billion vaccine baits have been safely and effectively distributed globally [3,9]. Only about two dozen documented cases of vaccine-induced rabies in both target and non-target species have been reported during this time, all of these have been linked to first-generation modified live viruses (MLVs), but not a single case in humans. This corresponds to an exceptionally low incidence rate in animals of 1 in 48 million vaccine doses [9,30–35]. Furthermore, only two SAEs involving temporary skin lesions in immunocompromised individuals are linked with a recombinant vaccine based on the vaccinia virus [36,37].

Rabies control strategies using ORVs targeting dogs should consider additional safety precautions than when targeting wildlife populations because of the close relationship between dogs and humans. Regular close interactions between humans and dogs may increase the likelihood of indirect and direct human contact with the vaccine virus. However, evaluating the safety for humans and the likelihood of human contact with the vaccine, based on available experimental and field data, is challenging. Previously recommended safety tests on non-human primates for ORVs [38] have been reconsidered due to ethical concerns, scientific advancements, and alternative methods. Non-human primates are not natural hosts for rabies and do not mimic the target wildlife species (such as foxes, raccoons, or dogs) for which ORVs are developed. Also, using primates did not improve safety prediction compared to small animal models (e.g., rodents) and did not lead to different safety conclusions. International bodies emphasize the “3Rs principle” (Replacement, Reduction, Refinement) to minimize animal use, and alternative models adequately address ORV safety. As a result, the CDC (Atlanta, USA) developed a Markov Chain Model to map possible exposure pathways (depending on the distribution system and targeted species) and to estimate the number of SAEs in humans based on the safety profile of the vaccine virus [10]. These studies demonstrate that the vaccine is safe (due to reverse genetic modification preventing the virus construct from reverting to virulence), with no SAE to target or non-target species or the environment. Furthermore, the distribution of SPBN GASGAS vaccine bait by the handout model for dogs minimizes the likelihood of accidental human exposure [29]. The absence of SAE in humans as estimated in the model are supported by previous large-scale ORV field trials with SPBN GASGAS where no SAE had been reported [39–41]. These field studies also showed the great acceptability of ORV, both by the veterinary authority, vaccinators, dog owners, and dogs [40,41]. Also, the operational effectiveness in the field was shown [41].

The recently published guidelines for Oral Vaccination of Dogs against Rabies by the Tripartite [6] supersedes technical information in previous World Health Organization foundational documents on oral rabies vaccination of dogs [42,43] which includes new recommendations on managing potential human contact with the vaccine virus. Due to the safety profile of SPBN GASGAS, the guidelines have been updated regarding the requirement for PEP following direct or indirect exposure to the vaccine virus. PEP is no longer required except in cases where the person is immunocompromised and if the vaccine exposure involves intramuscular, intraperitoneal, or intracranial inoculation and depending on the severity of the immune disorder. Scientific evidence confirms that even repeated exposures in humans, though extremely unlikely, would only result in an immune response, but not in any SAE.

This combination of safety, biological efficacy, and operational practicality has positioned SPBNGASGAS and ORV as a valuable adjunct to parenteral vaccination in achieving global rabies elimination targets. Nevertheless, despite this robust evidence base, certain data gaps and uncertainties remain. One area of uncertainty concerns the potential for horizontal transmission of vaccine virus between animals. ORVs are designed for oral administration to individual dogs, and available data indicate that the risk of inherent pathogenicity or onward transmission through licking, grooming, or scavenging is extremely low. However, systematic documentation of such events under field conditions is challenging. Continued molecular surveillance of rabies cases in animals is therefore recommended [44] to ensure early detection of any unexpected case, however improbable they may be.

## Conclusion

Since dogs and humans not only share the same environment but often even the same households, ORVs intended for dogs should meet more stringent safety requirements than vaccines distributed for wildlife [23,25]. ORV distribution for wildlife often occurs in rural habitats with lower human populations and where the likelihood of humans encountering vaccine baits is unlikely. Currently, SPBN GASGAS is the only commercially available ORV strain that meets all safety requirements as set out in WOAH’s Terrestrial Manual with a specific request for a risk assessment on human safety [25]. The suggested distribution method, hand-out and retrieve method reduces potential human exposures considerably; however, direct and indirect contact with the vaccine virus cannot be completely prevented. Fortunately, due to the safety profile of the vaccine construct SPBN GASGAS, it can be concluded that the risk for SAE in humans upon contact with the vaccine virus SPBN GASGAS is negligible. Considering the benefits of eliminating rabies at its source, by vaccinating the free-roaming dogs often not accessible by conventional vaccination methods by the injectable route, outweighs the identified negligible risks associated with the third-generation MLV vaccine strain, SPBN GASGAS.

## Supporting information

S1 Table Supplementary table—comparative overview of oral rabies vaccines. (PDF)
