# Supplementary material for: Human safety of SPBN GASGAS for oral rabies vaccination of dogs
Source: PLoS Negl Trop Dis. 2026 Jan 12;20(1):e0013866. doi: 10.1371/journal.pntd.0013866 (PMC12795385; doi:10.1371/journal.pntd.0013866)
Supplement: S1 Table — (PDF) [file pntd.0013866.s001.pdf]

| Vaccine strain | Vector | Vaccine generation | Type of vaccine | GMO[1] | Basis for attenuation | Tested in dogs | License for dogs | Residual pathogenicity | Genetic stability[2] | Vaccine induced rabies cases in animals | Adverse events in humans | Human risk assessment | Safety profile | References |
|---|---|---|---|---|---|---|---|---|---|---|---|---|---|---|
| **SPBN GASGAS** | **RABV** | **3rd** | **attenuated** | **Yes** | **Reverse genetics with multiple site-directed mutagenesis** | **Yes** | **Yes** | **extremely low** | **Yes** | **No** | **No** | **Yes** | **exceptional** | **[1-5]** |
| ERA G333 | RABV | 3rd | attenuated | Yes | Reverse genetics | Yes | No | extremely low | -[3] | - | - | No | very high | [6] |
| SAG2 | | 2nd | | No | Monoclonal selection mutant | Yes | No | extremely low | - | No | No | No | very high | [7-11] |
| SAD Bern | | 1st | | No | Serial passaging/clonal selection | N/A | No | moderate | - | Yes | No | No | high | [12] |
| SAD B19 | | 1st | | No | | Yes | No | moderate | Yes | Yes | No | No | high | [13-16] |
| VRG | Vaccinia backbone | N/A[4] | recombinant | Yes | Recombinant, expressing rabies glycoprotein | Yes | No | No | - | N/A | Yes | No | very high | [17,18] |
| AdRG1.3 | Human adenovirus backbone | | | Yes | | No | No | No | - | N/A | No | No | very high | [19-21] |

Supplementary File S2: Comparative Overview of Oral Rabies Vaccines

[1] GMO: genetically modified organism
[2] Full genome sequence analysis using next generation sequencing after serial passaging
[3] -: No information available
[4] N/A: not applicable

# References


1. Freuling CM, Eggerbauer E, Finke S, Kaiser C, Kaiser C, Kretzschmar A, et al. Efficacy of the oral rabies virus vaccine strain SPBN GASGAS in foxes and raccoon dogs. Vaccine. 2019; 37 (33): 4750-4757. doi:10.1016/j.vaccine.2017.09.093.

2. Vos A, Freuling C, Ortmann S, Kretzschmar A, Mayer D, Schliephake A, et al. An assessment of shedding with the oral rabies virus vaccine strain SPBN GASGAS in target and non-target species. Vaccine. 2018; 36 (6): 811-817. doi:10.1016/j.vaccine.2017.12.076.

3. Ortmann S, Kretzschmar A, Kaiser C, Lindner T, Freuling CM, Kaiser C, et al. In Vivo Safety Studies With SPBN GASGAS in the Frame of Oral Vaccination of Foxes and Raccoon Dogs Against Rabies. Front Vet Sci. 2018; 5: 91. doi:10.3389/fvets.2018.00091.

4. Freuling CM, Kamp VT, Klein A, Günther M, Zaeck L, Potratz M, et al. Long-Term Immunogenicity and Efficacy of the Oral Rabies Virus Vaccine Strain SPBN GASGAS in Foxes. Viruses. 2019; 11 (9). doi:10.3390/v11090790.

5. European Medicines Agency, Committee for Medicinal Products for Veterinary Use. CVMP assessment report for Rabitec (EMEA/V/C/004387/0000). London: EMA. 2017;

6. Bankovskiy D, Safonov G, Kurilchuk Y. Immunogenicity of the ERA G 333 rabies virus strain in foxes and raccoon dogs. Dev Biol (Basel). 2008; (131): 461–466.

7. EMEA. Rabigen SAG2. EPAR. Scientific discussion. London. 2004;. 26 p.

8. European Agency for the Evaluation of Medicinal Products. Committe for Veterinary Medicinal Products - European Public Assessment Report RABIGEN SAG 2. 2000;

9. Masson E, Cliquet F, Aubert M, Barrat J, Aubert A, Artois M, et al. Safety study of the SAG2 rabies virus mutant in several non-target species with a view to its future use for the immunization of foxes in Europe. Vaccine. 1996; 14 (16): 1506–1510. doi:10.1016/s0264-410x(96)00114-4.

10. Cliquet F, Gurbuxani JP, Pradhan HK, Pattnaik B, Patil SS, Regnault A, et al. The safety and efficacy of the oral rabies vaccine SAG2 in Indian stray dogs. Vaccine. 2007; 25 (17): 257–264. doi:10.1016/j.vaccine.2006.12.054.

11. Hsu AP, Tseng CH, Barrat J, Lee SH, Shih YH, Wasniewski M, et al. Safety, efficacy and immunogenicity evaluation of the SAG2 oral rabies vaccine in Formosan ferret badgers. PloS One. 2017; 12 (10): e0184831. doi:10.1371/journal.pone.0184831.

12. Wandeler AI, Capt S, Kappeler A, Hauser R. Oral Immunization of Wildlife Against Rabies. Concept and First Field Experiments. Rev Infect Dis. 1988; 10 (Supplement 4): S649-S653. doi:10.1093/clinids/10.Supplement_4.S649.

13. Estrada R, Vos A, Leon R de, Mueller T. Field trial with oral vaccination of dogs against rabies in the Philippines. BMC Infect Dis. 2001; 1: 23. doi:10.1186/1471-2334-1-23.

14. Vos A, Neubert A, Aylan O, Schuster P, Pommerening E, Müller T, et al. An update on safety studies of SAD B 19 rabies virus vaccine in target and non-target species. Epidemiol Infect. 1999; 123 (1): 165–175. doi:10.1017/s0950268899002666.

15. Neubert A, Schuster P, Müller T, Vos A, Pommerening E. Immunogenicity and efficacy of the oral rabies vaccine SAD B19 in foxes. J Vet Med B Infect Dis Vet Public Health. 2001; 48 (3): 179-183. doi:10.1046/j.1439-0450.2001.00440.x.

16. Vos A, Pommerening E, Neubert L, Kachel S, Neubert A. Safety studies of the oral rabies vaccine SAD B19 in striped skunk (Mephitis mephitis). J Wildl Dis. 2002; 38 (2): 428–431. doi:10.7589/0090-3558-38.2.428.

17. Maki J, Guiot AL, Aubert M, Brochier B, Cliquet F, Hanlon CA, et al. Oral vaccination of wildlife using a vaccinia-rabies-glycoprotein recombinant virus vaccine (RABORAL V-RGR®). A global review. Vet Res. 2017; 48 (1): 57. doi:10.1186/s13567-017-0459-9.

18. Cliquet F, Barrat J, Guiot AL, Caël N, Boutrand S, Maki J, et al. Efficacy and bait acceptance of vaccinia vectored rabies glycoprotein vaccine in captive foxes (Vulpes vulpes), raccoon dogs (Nyctereutes procyonoides) and dogs (Canis familiaris). Vaccine. 2008; 26 (36): 4627–4638. doi:10.1016/j.vaccine.2008.06.089.

19. Knowles MK, Nadin-Davis SA, Sheen M, Rosatte R, Mueller R, Beresford A. Safety studies on an adenovirus recombinant vaccine for rabies (AdRG1.3-ONRAB) in target and non-target species. Vaccine. 2009; 27 (47): 6619–6626. doi:10.1016/j.vaccine.2009.08.005.

20. Knowles MK, Roberts D, Craig S, Sheen M, Nadin-Davis SA, Wandeler AI. In vitro and in vivo genetic stability studies of a human adenovirus type 5 recombinant rabies glycoprotein vaccine (ONRAB). Vaccine. 2009; 27 (20): 2662–2668. doi:10.1016/j.vaccine.2009.02.074.

21. Fry TL, Vandalen KK, Duncan C, Vercauteren K. The safety of ONRAB(R) in select non-target wildlife. Vaccine. 2013; 31 (37): 3839-3842. doi:10.1016/j.vaccine.2013.06.069.
